# An online intervention relationship enhancement couple therapy program versus systematic desensitization on sexual performance anxiety in women undergoing infertility treatment with timed intercourse: An RCT

**DOI:** 10.18502/ijrm.v22i12.18062

**Published:** 2025-01-31

**Authors:** Maryam Afzal Dehkordi, Tahmineh Farajkhoda, Mahdi Khanabadi, Farimah Shamsi

**Affiliations:** ^1^Student Research Committee of Nursing and Midwifery School, Shahid Sadoughi University of Medical Sciences, Yazd, Iran.; ^2^Research Center for Nursing and Midwifery Care, Non-Communicable Diseases Research Institute, Department of Midwifery, School of Nursing and Midwifery, Shahid Sadoughi University of Medical Sciences, Yazd, Iran.; ^3^Department of Counseling, School of Education Sciences and Psychology, Allameh Tabataba'i University, Tehran, Iran.; ^4^Center for Healthcare Data Modeling, Department of Biostatistics and Epidemiology, School of Public Health, Shahid Sadoughi University of Medical Sciences, Yazd, Iran.

**Keywords:** Psychological interventions, Sexual health, Infertility, Women, Randomized clinical trial.

## Abstract

**Background:**

Sexual performance anxiety is common among infertility treatments. Psychological interventions are options for couples who do not tolerate psychiatric medications or like psychotherapy with pharmacotherapy.

**Objective:**

This study aimed to compare the effectiveness of relationship enhancement couple therapy program (RECT) vs. systematic desensitization (SD) on sexual anxiety.

**Materials and Methods:**

A parallel randomized clinical trial was conducted on 68 women with infertility in Shahrekord, Iran, in January 2022. They were randomly allocated to 8 weekly online interventions RECT or SD (control). The main outcome variable, sexual performance anxiety, was assessed via Davis's questionnaire at baseline, completion of the intervention (week 8), and follow-up (week 12). The secondary outcome variable spouse's satisfaction was assessed (weeks 8 and 12, via visual analog scale). Data from 29 women were analyzed in each group.

**Results:**

Intra-group comparison RECT and SD showed a significant decrease of sexual anxiety baseline compared to week 12 (p = 0.001, 0.001 for RECT and SD, respectively). No significant difference was observed between the 2 groups in baseline compared to week 12 (RECT vs. SD, p = 0.149). Time-group interaction of spouse's satisfaction showed a significant difference between RECT and SD (p = 0.010).

**Conclusion:**

Both RECT and SD were almost equally effective in decreasing sexual anxiety. RECT (couple relationship-centered) by empowering 10 interpersonal skills, and SD (personal-oriented) by learning step-by-step how fight-or-flight response is replaced by relaxation response, helping women perform more adaptive behaviors regarding sexual anxiety. RECT increased spouse satisfaction significantly compared to SD, indicating that males need to participate in positive relationship changes. The results may be applied by therapists and health policymakers.

## 1. Introduction

The relationship between stress and infertility has long been a subject of debate, with stress caused by assisted reproductive technologies being considered nearly as severe as the death of a loved one or divorce (1). Beyond the physical demands, psychological distress stemming from anxiety about treatment outcomes, frequent clinic visits, and fears of marital strain exacerbates this stress, potentially leading to sexual anxiety, dysfunction, and dissatisfaction in both partners (1, 2).

Timed intercourse, often synchronized with ovulation, can intensify stress, leading to sexual performance anxiety (SPA), where fear negatively affects sexual function. SPA, a common issue, affects 9–25% of men, causing premature ejaculation or erectile dysfunction, and 6–16% of women, leading to inhibited sexual desire and dysfunction. Couples dealing with infertility frequently lack the necessary skills to manage these challenges, making psychological interventions crucial (3, 4).

Research emphasizes the importance of psychological and counseling interventions, with evidence suggesting that cognitive-behavioral therapy and other psychological strategies are more effective than pharmacological treatments in increasing pregnancy rates among women with infertility (5). Techniques like neurofeedback (6), mindfulness (7), cognitive-behavioral therapy (8), and acceptance and commitment therapy (9) may eliminate sexual anxiety.

Relationship enhancement couple therapy (RECT), a couple-centered psychoeducational approach founded by Bernard Garney in 1962, has been effective in improving intimacy, sexual satisfaction, and marital adjustment. RECT combines elements of client-centered theory, learning and behavior change theories, interpersonal theory, and psychodynamic theory, focusing on skill training in areas such as empathy, conflict management, and problem-solving (10, 11).

Systematic desensitization (SD), a behavioral therapy technique for anxiety disorders, is another approach that has been used to address sexual anxiety. This technique involves teaching relaxation methods, creating a hierarchy of anxiety-inducing stimuli and gradually exposing the client to these stimuli while employing relaxation techniques. Studies have shown that SD is significantly effective in reducing sexual dysfunction and anxiety (12).

Given the limited comparative studies on SPA in couples affected by infertility, particularly those lacking online formats, control groups, or follow-up plans, this study seeks to address this gap (13–15). In a study that found women affected by SPA who received couple therapy after regular desensitization, the authors suggested that future studies should compare the effects of group SD in women vs. group couple therapy (12). The study question arises as to which of the 2 interventions, RECT vs. SD (control group), reduces sexual anxiety in women undergoing infertility treatment with timed intercourse with greater continuity and spouse's satisfaction.

This study aimed to compare RECT vs. SD on SPA in women undergoing infertility treatment with timed intercourse.

## 2. Materials and Methods

A parallel randomized clinical trial study was conducted on women suffering from infertility. According to a study by Rabeipour et al. taking into account the standard deviation of sexual relationship anxiety scores in intervention and control groups, respectively 6.2 and 7.45 and 95% confidence level and 80% power, a minimum difference of 5.2 and 20% of sample attrition, 34 samples in each group were calculated (16). The first researcher carried out the recruitment and sampling for the study.

Participants were women suffering from infertility referring to Hazrat Zahra Infertility Treatment Clinic in Shahrekord, Iran in 2022 who expressed sexual intercourse anxiety. Inclusion criteria included Iranian language, having a medical record of primary infertility in the mentioned center, having sexual intercourse anxiety based on the interview and sexual anxiety scale questionnaire, having a smartphone and internet literacy, having a sexual relationship with her husband, willingness to participate in the study and satisfaction of the husband for the woman's participation in the study. Exclusion criteria included pregnancy, Ovarian Hyper Stimulation Syndrome, having neurological and psychiatric diseases based on their medical report and women's self-report, taking psychiatric drugs based on infertility records, misfortune events such as the death of near relatives in the last 2 months, family quarrels in the last month, living in the location with no access to the internet, and participation in psychological counseling sessions or similar studies in the last 6 months.

For sequence generation, eligible women were randomly (simple randomization) assigned to one of the 2 investigated groups based on random allocation software. Allocation of the participants to 2 groups was done by a biostatistician (the last investigator).

125 women undergoing infertility treatment were recruited and assessed by the first investigator, A.M. subsequently, 68 eligible women were identified to enter the study and were randomized into 2 groups (n = 34/each group). Allocation concealment was done by telephonic method (both the first researcher who did the sampling and the women participating in the study were concealed to the type of allocation).

The first investigator assigned participants to interventions. Women were invited to 2 separate WhatsApp groups to receive videos related to some necessary explanations, regarding, how to participate in sessions and complete the questionnaires. The electronic link of the questionnaires was sent to them before the intervention. The study was conducted only on women in both groups. The husbands were indirectly asked about their satisfaction with the intervention. In the RECT group, the content of sessions were provided based on couple therapy. The first and second investigators answered the women's questions in WhatsApp.

### Intervention and measurements

Blinding was impossible because the women were informed about the type of intervention. Data collection in both groups was done using electronic links to questionnaires via Porsline.

#### Demographic information

It included the age of the couple, education of the couple, occupation of the couple, length of marriage, time of infertility diagnosis, type of infertility treatment, cause of infertility, and duration of treatment. It was assessed at baseline.

#### The Davis sexual anxiety questionnaire (primary outcome)

It is a self-report tool with the 18-item for measurement of SPA (17). Answering the questions was based on a 5-point Likert scale, scored from 1–5. The scoring of the questionnaire included 18–43 with low sexual anxiety, 44–64 with moderate sexual anxiety, and 65–90 with high sexual anxiety. The reliability of the internal consistency type was reported by Cronbach's alpha method on a scale of 0.93. Questionnaire scoring for questions 4, 5, 8, 9, 10, 12, 14, 15, 16, 17, 18 was done reversely (18). In Iran, Ghafari and Roshan have also evaluated this questionnaire. Convergent validity ranged from 0.50–0.79, and divergent validity ranged from -0.40 to -0.62 (p < 0.05). The questionnaire's Cronbach's alpha was 0.93, and the correlation coefficient between the 2 halves of the test was 0.77 (19). The questionnaire was used for measurement of the main outcome variable SPA at baseline, week 8, and week 12.

#### Spouse's satisfaction (secondary outcome)

Measuring the spouse's satisfaction with the intervention because of the wife, which was measured with a question on a 10-point visual analog scale from 1–10 (20). The intra-class coefficient was 0.95 (21). The questionnaire was used for the measurement of the secondary outcome variable spouse's satisfaction in week 8 and week 12.

### Intervention

Interventions were performed for both groups separately in the Skyroom through online lectures, PowerPoint, clips, text, photos, group chat, and question and answer in 8 sessions for 60-min by the first investigator under the supervision of the second (psychologist) and third investigator (Ph.D. in family counseling) based on table I and II. Intervention packages were prepared based on scientific resources applicable to SPA, including session strategies, assignments, and content for RECT (Table I) (11, 13, 14, 22), and SD (Table II) (15, 23, 24). SMS reminders were sent to both groups before each session. In addition, women asked their questions on WhatsApp. At the beginning of each session, a summary of the previous session was presented, and women's assignments were evaluated.

**Table 1:** Strategies and the content of counseling sessions with the RECT

**Sessions **	**Strategies**	**The content of the sessions**	**Homework**
**1**	• Formation of therapeutic rapport • Group's regulations • Targeting	• Explanation of goals and objectives • Discussion on causes of infertility and kinds of infertility treatment • Assessment of women's experiments and concerns about sexual anxiety during infertility treatment	• Expressing the effect of infertility on the relationship with the spouse • Accurate disclosure of anxiety in relation to spouse
**2**	• The teaching of discussion and negotiation skills	• Including encouragement, modeling, inhibitory modeling, guidance, and preparation • Providing examples of experiencing SPA	• Transferring the training to the husband • Discussing the effect of infertility on sex
**3**	• Teaching empathic skills	• Using the skill of deep empathy and intimate dialogue for a deeper understanding of the spouse and the relationship between the couple • Self-exploration and self-disclosure encourage them to express what they experience in the course of infertility treatment	• Writing a letter from his wife to himself • Teaching the wife
**4**	• Teaching expressive skills	• Cultivating the spirit of participation and cooperation • Decreasing defensiveness in the relationship • Trying to find a solution to the problem	• Teaching exercises with spouse as a team • Preparing a list of solutions proposed by couples to solve SPA
**5**	• Teaching management and conflict resolution skills	• Increasing the ability to control emotions and manage conflict situations • Providing creative solutions based on the expectations of the parties from the relationship • Find a solution to the problem	• Applying this skill in 3 cases of solvable conflicts in their relationship
**6**	• Teaching the skill of sexual enrichment	• Explaining the difference between sexual satisfaction and sexual pleasure • Expansion of nonerotic and erotic sex games • Having realistic expectations for sex	• Exercises related to expanding the range of sexual relations
**7**	• Teaching facilitation and change skills	• Acting quickly and confidently to change behavior • Be patient to make changes in the relationship	• Writing a letter to themselves from 1 yr later by couples expressing the desired changes that have occurred in their relationship
**8**	• Teaching the skill of generalization and maintenance	• Practicing the skill of enriching the relationship without considering the amount of change in the spouse • Monitoring of SPA changes	• Examining the ways to increase the durability of the changes and the factors hindering the change • Providing solutions to increase the sustainability of changes
RECT: Relationship enhancement couple therapy, SPA: Sexual performance anxiety			

**Table 2:** Strategies and the content of counseling sessions with the SD

**Sessions**	**Strategies**	**The content of the cessation**	**Homework**
**1**	• Formation of therapeutic rapport • Group's regulations • Targeting • Psychoeducation for anxiety	• Explanation of goals and objectives • Discussion on causes of infertility and kinds of infertility treatments • Assessment of women's experiments and concerns about sexual anxiety during infertility treatment • Difference between fear and anxiety • Explaining the strategies to reduce anxiety, their role and importance	• What is paradox experienced in sexual activity during infertility treatments? • How does a couple feel SPA? • What did they do for SPA?
**2**	• Establish hierarchy: ∘ Organizing and finalizing the anxiety	• Hierarchy list based on anxiety-activating experiences, situations, or events • Rank anxiety-provoking events from mildest to most severe regarding infertility and SPA	• Making a list of anxiety-provoking events in sex • Sort the list of anxiety-provoking events • Rating SPA for a couple
**3**	• Progressive muscle relaxation: ∘ Getting to know the Jacobsen relaxation	• Getting to know the muscles	• Practicing mental imagery regarding anxiety-provoking situations • Making a note of mental images
**4**	• Deep breathing technique: ∘ Abdominal relaxation	• Teaching relaxation techniques	• Relaxation practice at home according to the schedule
**5**	• 4, 7, 8 technique	• Teaching 4, 7, 8technique, and its benefits on SPA	• Relaxation practice at home according to the schedule
**6**	• Guided visualization: A calm place • Imagining the anxiety-causing event from mild to severe	• Teaching the use of visualizing an anxiety-provoking event	• Visualizing anxiety-provoking events in order from mild to severe, taking notes of the results
**7**	• Concentration techniques: ∘ Reverse counting	• Emphasizing the role of concentration on anxiety • Explain the difference between feelings, thoughts, emotions, and behavior	• Doing and taking notes of the results
**8**	• Real exposure	• Teaching how to face reality and how to overcome anxiety	• Exposure to a real anxiety-provoking event • Control the situation using relaxation techniques • Note the results
SD: Systematic desensitization, SPA: Sexual performance anxiety			

### Ethical Considerations

The study was approved by the Ethics Committee of Shahid Sadoughi University of Medical Sciences, Yazd, Iran (Code: IR.SSU.REC.1400.160 on 2021/11/17) and registered in the Iranian Registry of Clinical Trials 2021/12/25 with the code IRCT20211127053188N1, prospectively. All women signed the informed consent.

### Statistical Analysis

After collecting the data, they were coded and entered into SPSS version 15 and then analyzed using appropriate tests. Qualitative and quantitative data were reported using descriptive statistics according to the frequency distribution table, mean, and standard deviation. Kolmogorov-Smirnov test and Shapiro-Wilks test were used to check the normality of the data. Fisher's exact test for data with < 20% was performed for demographic data analysis. Friedman test, Mann-Whitney test, and Independent *t* test were applied to the effect of the primary outcome variable SPA with repeated measures were used. Mann-Whitney and Wilcoxon's tests were used to report the secondary outcome variable, and the spouse's satisfaction with the intervention. A significance level of 0.05 was considered in all tests.

## 3. Results

**Figure 1:**
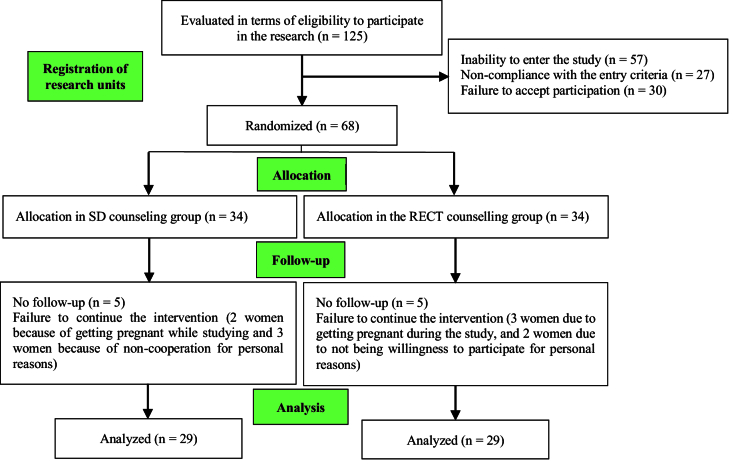
CONSORT flowchart of the research plan.

Initially, 125 women undergoing infertility treatment were assessed by the first investigator. Subsequently, 68 eligible women were identified to enter the study and then randomized into 2 groups (34 in each group). Among them, 5 women in the RECT group (3 women due to getting pregnant during the study, and 2 women due to not being willing to participate for personal reasons) and 5 in the SD group (control) (2 women because of getting pregnant while studying and 3 women because of non-cooperation for personal reasons) were excluded. Preventing their continued participation in the study. Finally, the data of 58 participants across the 2 groups were analyzed (Figure 1).

The results showed that no statistically significant difference was observed in the demographic variables (Table III).

The main outcome variable of sexual relationship anxiety indicated that intra-group comparison of RECT and SD showed a significant decrease in scores of sexual anxiety at baseline compared to week 12 and baseline, respectively. No significant difference was observed between the 2 groups in baseline compared to week 12 (Table IV).

The secondary outcome variable of spouse satisfaction was high in both RECT and SD without intergroup significant difference. Time-group interaction changes showed a significant difference between RECT and SD (Table V).

**Table 3:** Comparing the frequency of demographic variables in 2 groups

**Variables**		**Groups**		**P-value**
		**RECT**	**SD**	
**Woman's job**				
	**Housewife**	21 (72.5)	26 (89.7)	0.272
	**Self-employment**	5 (17.25)	2 (6.9)	
	**Employment**	3 (10.3)	1 (3.4)	
**Spouse's job**				
	**Self-employment**	22 (75.9)	22 (75.9)	0.990
	**Employment**	7 (24.1)	7 (24.1)	
**Woman's education**				
	**Under diploma**	3 (10.3)	3 (10.3)	0.472
	**Diploma**	10 (34.5)	15 (51.7)	
	**Associate degree**	2 (6.9)	3 (10.3)	
	**Bachelor's degree and above**	14 (48.3)	8 (27.7)	
**Spouse's education**				
	**Under diploma**	5 (17.2)	3 (10.3)	0.898
	**Diploma**	15 (51.7)	16 (55.1)	
	**Associate degree**	21 (3.4)	2 (6.9)	
	**Bachelor's degree and above**	8 (27.7)	8 (27.7)	
**Causes of infertility**				
	**Female causes**	22 (75)	17 (58.6)	0.357
	**Male causes**	3 (10.7)	3 (10.3)	
	**Both causes**	4 (14.3)	9 (31.1)	
Data presented as n (%), Fisher's exact test. RECT: Relationship enhancement couple therapy program, SD: Systematic desensitization				

**Table 4:** Comparing the mean score of sexual anxiety in 2 groups at baseline, week 8, and follow up

**Variables**			**Groups**		**P-value**
			**RECT**	**SD**	
Sexual anxiety*					
	**Baseline**		53.76 ± 5.9 48 (9.5)	55.45 ± 5.9 49 (10)	0.299
	**Week 8**		37.83 ± 7.4 38 (12)	40.44 ± 6 42 (8.5)	0.216
	**Follow-up week 12**		37.62 ± 7.3 39 (12.5)	41.21 ± 6.4 41 (8)	0.091
**The repeated measurements**		**F**	245.34	238.34	
		P-value**	< 0.001	< 0.001	—
**Score changes**					
	In the baseline phase and completion of the intervention in week 8***		-7.55 ± 3.65	-7.92 ± 4.26	0.869
	In the baseline phase and completion of the intervention in week 12***		-7.75 ± 3.68	-7.32 ± 4.05	0.671
	At the end of the intervention in the 8 ${\;}^{\text{th}}$ wk and the end of the study in the 12 ${\;}^{\text{th}}$ wk***		-0.20 ± 2.45	0.60 ± 2.57	0.277
*Data presented as Mean ± SD, Median (interquartile range), Mann-Whitney test, **Friedman test. ***Data presented as Mean ± SD, Independent *t* test. RECT: Relationship enhancement couple therapy program, SD: Systematic desensitization					

**Table 5:** Comparing the mean score of spouse satisfaction with intervention in 2 groups at week 8 and follow up

**Variables**		**Groups**						P-value*
		**RECT**			**SD**			
	**Completion of the intervention in week 8**	6.07 ± 1.27	6	2	6.31 ± 1.33	6	2	0.440
	**Completion of the study in week 12**	6.62 ± 1.01	6	1	6.13 ± 1.05	6	2	0.087
**Wilcoxon test**	P-value**	0.002			0.387			–
**Score changes at the end of the intervention in the 8 ${\;}^{\text{th}}$ wk and the end of the study in the 12 ${\;}^{\text{th}}$ wk**		0.55 ± 0.78			-0.17 ± 1.10			0.010
Data presented as Mean ± SD, *Mann-Whitney test, **Wilcoxon test. RECT: Relationship enhancement couple therapy program, SD: Systematic desensitization								

## 4. Discussion 

Both RECT and SD were almost equally effective in decreasing sexual anxiety. RECT increased spouse satisfaction significantly in comparison to SD. Assessment of the main outcome variable of sexual relationship anxiety showed that both RECT and SD interventions were equally able to reduce sexual anxiety. Since RECT and SD interventions were not compared in previous similar studies, the discussion is reported in a separate study.

In a study, the use of RECT reduced sexual anxiety scores (25). Another study revealed that prevention and relationship enhancement programs can improve couple's relationships and elevate their knowledge and skills (26). The effect of relationship enhancement training on the quality of marital relationships. Teaching the relationship enhancement approach can significantly reduce job stress and increase the quality of marital relationships (13).

A research also reported that marital enrichment programs indicated promising effects on sexual satisfaction and sexual function in females with disabilities (27). Based on one study, couple therapy based on the relationship enrichment method had a significant effect on sexual function and intimacy in the this group in compare with control group in all 3 times of follow up (22). Another research showed that sexual enhancement group couples therapy is an effective intervention for treatment of low sexual desire or frequency complaints in distressed couples. The largest improvement and effect sizes were observed in total satisfaction with one's sex life from the baseline to the end of intervention and 6-month follow up (28). Another research showed that both online and face-to-face sexual intimacy enhancement training programs can improve sexual intimacy and sexual satisfaction in pregnant women (29). A study reported that a marital relationship enrichment program can improve marital intimacy and marital and sexual satisfaction in infertile couples (30). A clinical trial that examined the effect of a relationship enhancement intervention on enhancement of marital intimacy in couples found that it positively influences on marital intimacy, sexual function, and sexual motivation in spouses (31).

In a descriptive-analytic study titled “Enriching the Spouse Relationship in the RE model", the theoretical structure, educational skills, techniques, methods, and procedures of RE treatment are clarified, then its advantages and disadvantages are discussed. Given the importance of cultural differences in counseling and psychotherapy work, this study has reached noteworthy points about the effectiveness and level of fitness in Iranian-Islamic culture in counseling with spouses and families (25).

Studies show that SD is effective in reducing anxiety (32, 33). Systematic virtual reality desensitization has also been used to treat psychogenic erectile dysfunction in a pilot study, and the results suggest that it is a potentially effective and safe treatment option for men with psychogenic erectile dysfunction (34).

In line with the results of this study, research reported that the SD program for women's sexual anxiety, improved marital adjustment, and sexual anxiety is significantly reduced. In addition, the perception of sexual irresponsibility of female partners was significantly reduced (12). SD is significantly associated with a decrease in the mean scores of state anxiety and fear of depth, as well as an increase in interest in continuing sports participation compared to the control group (35). Another study revealed that modified SD techniques have been much more effective in eliminating sexual dysfunction and reducing associated sexual and social anxiety compared to similar conditions (36).

The second outcome variable of this study was spouses satisfaction from the intervention. Time-group interaction changes showed a significant difference between RECT and SD, with more scores in RECT. It seems that the reason for this finding is that RECT is a couple-oriented method, and SD is an individual method. In RECT, men's participation in homework and learning skills leads to better resolution of marital conflicts and their feeling more empathy with women suffering from infertility. As a result, men also feel more satisfied in sexual relations (37, 38). Couples in difficult situations need emotional expression, which means the ability to dialogue, listen, openness, cooperate together as a team, and better relationship (39).

In a study on the effect of couple-oriented counseling on the intimacy of couples suffering from infertility, the effective role of this method in decreasing sexual anxiety has been supported by increasing the awareness of couples regarding appropriate communication between spouses. The results showed that counseling with couples, particularly in critical life situations, can promote their relationship during infertility treatment and prevent emotional divorce (40). A case study in Ardabil showed the effect of the sexual enrichment training program on the sexual satisfaction of couples, the training of the sexual enrichment program has been effective in increasing the level of sexual satisfaction in couples (13).

Designing a parallel clinical trial with 2 different short-term interventions (one centered on the individual and the other centered on the relationship between husband and wife) as well as designing and introducing a short-term intervention package for SPA in women undergoing infertility treatment with timed intercourse, assessing spouses satisfaction (not reported in similar studies) and designing an online intervention are strengths of this study that can be useful for other researchers. The application of the results in the clinical context is related to its usefulness in the integration of care guidelines and treatment plans for these women, as well as by sex therapists, health providers, and health policymakers.

### Strengths and limitations

One of the limitations of this study was the occasional disconnection of the internet during some sessions. To compensate for this problem, with the agreement of the women, the duration of the session was considered longer so that the entire content of that session could be covered. The impossibility of blinding was another limitation of the study because the women of each group were informed about the type of intervention.

## 5. Conclusion

Both RECT and SD were almost equally effective in decreasing sexual anxiety. RECT (couple relationship-centered) by empowering interpersonal 10 skills, and SD (personal-oriented), by learning step-by-step how fight-or-flight response is replaced by relaxation response, helping women perform more adaptive behaviors regarding sexual anxiety. RECT increased spouse satisfaction significantly compared to SD, indicating that males need to participate in positive relationship changes. Since couples affected by infertility need to learn new techniques to overcome their critical condition in their marital life during infertility treatment. The results can be applied by therapists and health policymakers for treatment process in infertility treatment centers.

##  Data Availability

Data supporting the findings of this study are available from the corresponding author.

##  Author Contributions

M. Afzal Dehkordi and T. Farajkhoda designed the study and conducted the research, and interpreted the results. T. Farajkhoda, M. Afzal Dehkordi, and F. Shamsi monitored, evaluated, and analyzed the results of the study. M. Khanabadi designed and supervised counseling sessions. All authors approved the final manuscript and take responsibility for the integrity of the data.

##  Conflict of Interest

The authors declare that there is no conflict of interest.
